# Plasma circulating tumor DNA as a potential tool for disease monitoring in head and neck cancer

**DOI:** 10.1002/hed.25563

**Published:** 2018-12-15

**Authors:** Matthew Egyud, Praveen Sridhar, Anand Devaiah, Emiko Yamada, Stefanie Saunders, Anders Ståhlberg, Stefan Filges, Paul M. Krzyzanowski, Irina Kalatskaya, Wei Jiao, Lincoln D. Stein, Scharukh Jalisi, Tony E. Godfrey

**Affiliations:** ^1^ Department of Surgery, Boston Medical Center Boston University School of Medicine, Boston, MA, USA; ^2^ Department of Otolaryngology and Head and Neck Surgery, Boston Medical Center Boston University School of Medicine, Boston, MA, USA; ^3^ Sahlgrenska Cancer Center, Department of Pathology and Genetics, Institute of Biomedicine Sahlgrenska Academy at University of Gothenburg Gothenburg Sweden; ^4^ Wallenberg Centre for Molecular and Translational Medicine University of Gothenburg Gothenburg Sweden; ^5^ Department of Clinical Pathology and Genetics Sahlgrenska University Hospital Gothenburg Sweden; ^6^ Ontario Institute for Cancer Research Toronto Ontario Canada; ^7^ Department of Otolaryngology and Head and Neck Surgery Beth Israel Deaconess Medical Center, Boston, MA, USA

**Keywords:** cancer biomarker, cancer diagnostics, cell‐free DNA, circulating tumor DNA, liquid biopsy

## Abstract

**Background:**

Recommendations for perioperative therapy in head and neck cancer are not explicit and recurrence occurs frequently. Circulating tumor DNA is an emerging cancer biomarker, but has not been extensively explored for detection of recurrence in head and neck cancer.

**Methods:**

Patients diagnosed with head and neck squamous cell carcinoma were recruited into the study protocol. Tumors were sequenced to identify patient‐specific mutations. Mutations were then identified in plasma circulating tumor DNA from pre‐treatment blood samples and longitudinally during standard follow‐up. Circulating tumor DNA status during follow‐up was correlated to disease recurrence.

**Results:**

Samples were taken from eight patients. Tumor mutations were verified in seven patients. Baseline circulating tumor DNA was positive in six patients. Recurrence occurred in four patients, two of whom had detectable circulating tumor DNA prior to recurrence.

**Conclusion:**

Circulating tumor DNA is a potential tool for disease and recurrence monitoring following curative therapy in head and neck cancer, allowing for better prognostication, and/or modification of treatment strategies.

## 1 INTRODUCTION

Head and neck cancer encompasses a heterogenous group of malignancies that occur in several locations along the aerodigestive tract, with squamous cell carcinoma as the most common. In 2018, head and neck cancers are estimated to affect approximately 51 000 people, leading to over 10 000 deaths in the United States.^1^ Head and neck cancer is more prevalent globally, affecting over 500 000 people and leading to death in more than half of these individuals.^1^ Additionally, there has been an increase in the incidence of human papillomavirus associated head and neck cancers. This entity of human papillomavirus positive head and neck cancers accounts for nearly half of the head and neck squamous cell carcinomas diagnosed in the United States, and is associated with an improved prognosis.^2^

Advanced head and neck squamous cell carcinomas are often diagnosed with regional disease, but treatment with intent to cure remains the goal for nearly all patients who do not have distant metastases.^3^ Following curative therapy, patients undergo post‐treatment surveillance for recurrence which includes serial examinations, endoscopy, computed tomography, magnetic resonance imaging, and positron emitted tomography‐computed tomography. Clinical suspicion of recurrence based on these exams can lead to endoscopies, biopsies, and other interventions to ascertain confirmation of disease.

Depending on the tumor characteristics, locoregional recurrence can occur at a relatively high rate and may be difficult to diagnose following treatment. Recurrences may be difficult to identify with current methods of surveillance due to tissue changes from curative therapy and reconstruction methods.^4^ Recurrence may be treated with different modalities, depending on tumor extent, location, and other characteristics. If salvage surgery is indicated, this can often be technically demanding in the setting of tumor recurrence.^3^, ^5^

Combination therapy is typically considered for patients with tumors having specific anatomic and pathologic findings at the time of presentation or during the course of their primary mode of therapy. In some instances, de‐escalation of treatment can be considered, and this is an area of active investigation. The clinical challenges of determining de‐escalation, adjuvant therapy, and early recurrence detection may be addressed in part by a sensitive biomarker that allows for the biochemical detection of disease, at a timepoint where conventional methods may not yield reliable results or may subject a patient to potentially unnecessary and invasive tests.

Circulating tumor DNA in plasma has been studied increasingly as a potential biomarker for cancer detection, monitoring of disease burden, and tumor response monitoring.^4^, ^6^, ^7^, ^8^, ^9^, ^10^, ^11^, ^12^, ^13^, ^14^, ^15^, ^16^, ^17^, ^18^, ^19^, ^20^, ^21^, ^22^, ^23^, ^24^, ^25^, ^26^, ^27^, ^28^, ^29^, ^30^ Despite this interest, circulating tumor DNA remains challenging to detect and to quantify as it represents a small fraction of total plasma circulating cell‐free DNA. Novel PCR‐based sequencing approaches have improved our ability to detect rare tumor mutations present in circulating tumor DNA against the total circulating cell‐free DNA background.^13^, ^14^

There has been a paucity of studies examining the utility of circulating tumor DNA as a precision medicine application, which may inform clinicians on prognosis and surveillance for head and neck squamous cell carcinoma.^7^, ^18^ The objectives of this study are to quantify patient‐specific changes in circulating tumor DNA following curative therapy and to examine the potential role of circulating tumor DNA in treatment monitoring and recurrence detection in head and neck squamous cell carcinoma patients.

## 2 METHODS

### 2.1 Patient recruitment

Institutional Review Board approval was obtained prior to the start of this study. Prior to enrollment in the study, patients provided informed consent for use of their tissues, blood samples, and data for the purpose of this research. Eligible patients included those of all stages (I‐IV) undergoing medical and/or surgical treatment for all histological subtypes of pathologically proven head and neck cancer. Patients meeting inclusion criteria underwent blood draws (EDTA tubes) just prior to surgical resection or definitive therapy. Tumor tissue was obtained either from resected specimens or from diagnostic biopsy specimens for DNA isolation. Patients were approached for blood draws at routine post‐operative visits conducted 3 weeks, 6 weeks, 3 months, 6 months, and 1 year following treatment. Blood samples collected at each time point were separated into plasma and a leukocyte containing buffy coat. DNA isolated from the buffy coat leukocytes was used as a reference, while DNA isolated from plasma contained the circulating tumor DNA of interest.

### 2.2 Blood processing

Plasma was separated within 1 hour of phlebotomy. Whole blood was centrifuged at 4°C for 10 minutes at 1600 RCF. The separated plasma component was again centrifuged at 3600 RCF for 10 minutes to ensure that the plasma was acellular. Plasma was then aliquoted and frozen at −80°C until DNA isolation. Cellular content, including mostly leukocytes and a small component of erythrocytes, was removed from the original centrifuged sample and frozen at −80°C until reference DNA isolation.

### 2.3 Extraction of DNA from plasma and buffy coat

Frozen plasma and buffy coat samples were thawed at room temperature and DNA was extracted using the QIAamp Circulating Nucleic Acid Kit (Qiagen) vacuum protocol. Samples were eluted in 50 uL of buffer AVE (RNAse free water with 0.04% Sodium Azide, Qiagen). For DNA extraction from buffy coats, 200 uL of buffy coat from one blood sample for each patient was used. Samples were eluted in 200 uL of buffer EB (10 mM Tris‐CL, pH 8.5, Qiagen). DNA concentrations were measured by fluorimetry (Qubit dsDNA HS Assay Kit, ThermoFisher Scientific). Isolated DNA was stored at −20°C. If necessary, circulating tumor DNA was concentrated to approximately 5 uL using a Vivacon 500 column with 30 000 molecular weight cut‐off (30 kDa) (Sartorius).

### 2.4 Extraction of DNA from tumor samples

Formalin‐fixed paraffin‐embedded tissue specimens were sectioned using a microtome and stained with hematoxylin and eosin for pathologic verification of >50% tumor content. Additional tissue was sectioned (a total of 100‐200 μM) and used for DNA extraction using the QIAamp DNA formalin‐fixed paraffin‐embedded Tissue Kit (Qiagen). Samples were eluted in 100 uL buffer AE (10 mM Tris‐Cl, 0.5 mM EDTA, pH 9.0, Qiagen) and DNA concentration was measured using fluorimetry (Qubit dsDNA HS Assay Kit, ThermoFisher Scientific). Samples were diluted in buffer EB and stored at −20°C for further use.

### 2.5 Identification of mutations in primary tumor samples

DNA from primary tumor samples and matched normal DNA from buffy coats were sequenced using whole‐exome or targeted next‐generation sequencing panels and mutations were identified using previously published methods.^13^, ^14^ The Mutect2 and Strelka algorithms were used to generate variant call files consisting of several somatic mutations.^31^, ^32^ Multiple mutations were selected for each tumor based on the total number of mutations identified, the variant allele frequency, the presence of the mutation in the Catalogue of Somatic Mutations in Cancer database, mutation location (exonic, intronic, etc), and predicted functional impact. Mutation sites were visually inspected using the Integrated Genome Viewer software (Broad Institute, Cambridge, Massachusetts) to filter mutational calls resulting from likely sequencing errors. All selected mutations were verified in the tumor using a Simple, Multiplexed, PCR‐based barcoding of DNA for Sensitive mutation detection using Sequencing (SiMSen‐Seq). This ultra‐sensitive sequencing method is described below.

### 2.6 Identification of mutations in plasma DNA

Mutations in plasma DNA were detected using SiMSen‐Seq, a next‐generation sequencing method that employs molecular barcodes for detection of mutant alleles at 0.1% frequency or higher.^13^, ^14^ SiMSen‐Seq is a PCR‐based next‐generation sequencing library construction approach that uses a random 12 base oligonucleotide, that is, molecular barcodes, to tag each individual DNA target strand in the early PCR cycles of library construction. Individual sequence reads that originate from the same target DNA molecule can therefore be identified and grouped into barcode families. In the absence of polymerase and sequencing errors, every read from a barcode family is theoretically identical. Reads that result from polymerase and sequencing error can be identified and discounted, as they occur only in a subset of the raw reads from an individual barcode family. Consequently, barcoding greatly reduces background sequencing noise and enables rare variant allele detection.^13^, ^14^

### 2.7 SiMSen‐Seq library creation and sequencing

SiMSen‐Seq assays were designed to target selected tumor mutations for each sample and subsequently tested and validated using previously described methods.^13^, ^14^ All target regions except for one assay were kept below 80 bp (short assays). Custom libraries were created using 25‐50 ng of tumor or plasma DNA and assays designed to capture each tumor's selected mutations. These libraries were then sequenced with an Illumina MiSeq sequencer using single‐end reads with 115‐130 cycles depending on amplicon length. Sequencing data files were analyzed using a custom program called Debarcer to group raw reads into barcode families, generate consensus reads for each barcode family, and plot minor allele frequency at each base in the amplicon.^13^ Variants were reported in consensus sequences if they composed 100% of the reads in families with 10‐20 reads, or at least 90% of reads in families with >20 reads.^12^

A plasma DNA sample was considered positive if any previously called patient‐specific mutation of interest was present above background noise, and with an alternative allele frequency of 0.05% or above. Patients with multiple mutations in the tumor required confirmation of one or more mutations in plasma to be considered positive.

## 3 RESULTS

### 3.1 Primary tumor and baseline plasma circulating tumor DNA detection and patient characteristics

**Figure 1:**
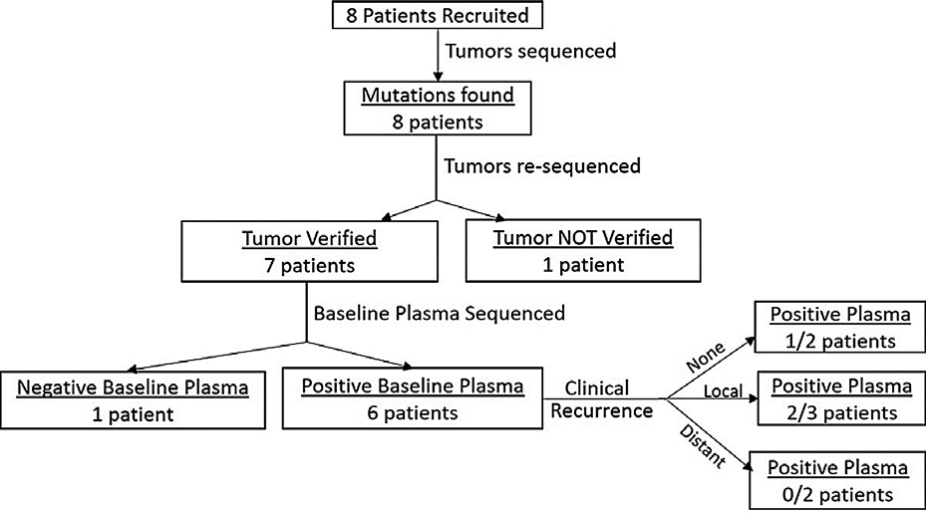
Consort diagram for eight patients diagnosed with head and neck cancer who were enrolled in this study

Consent was obtained from eight patients, of whom seven patients had their tumors resected and one had tissue diagnosis from a biopsy. Pathological staging was completed for all patients; there was one stage I tumor, six stage IVA tumors, and one stage IVB tumor. Tumors from all eight patients underwent targeted sequencing and mutations were identified in all samples. A visual summary of this workflow is provided in Figure 1.

**Figure 2:**
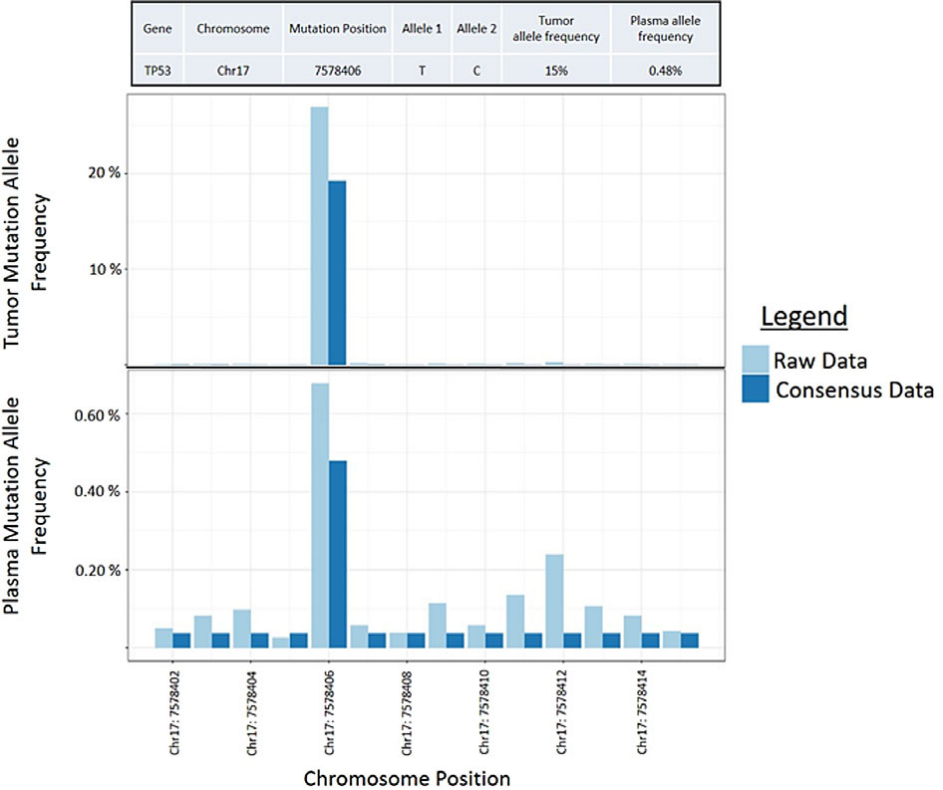
Raw positive result for plasma circulating tumor DNA. The baseline plasma sample in patient HN01 is represented in the bottom plot compared to the actual mutational frequency following correction by Debarcer (consensus data). This patient had a missense mutation at position chr17:7578406 in the tumor DNA (top) corresponding with the same mutation seen in the plasma circulating tumor DNA (bottom) [Color figure can be viewed at wileyonlinelibrary.com]

In total 42 tumor mutations were identified with tumor allele frequencies ranging from 5% to 30%. Plasma sequencing assays were designed for 28 of the 42 (67%) identified tumor mutations and were designed for at least two mutations from each of the eight tumors (range 2‐8 assays per patient). Tumor DNA was then re‐sequenced using SiMSen‐Seq to verify the presence of the selected mutations. A representative example of SiMSen‐Seq output data for tumor DNA and corresponding plasma circulating tumor DNA mutations can be seen in Figure 2. There were 22 mutations that verified in seven patients (two to seven mutations per patient). One patient had no verified mutations, and was therefore eliminated from further study (HN07). Baseline plasma samples were collected just prior to surgical resection. Variant allele frequencies observed in baseline plasma samples for each mutation are listed in Supporting Information Table S3. Following resection, four patients received adjuvant chemoradiation therapy. Longitudinal samples were collected from 3 weeks post‐therapy to as far as 18 months post‐therapy. Clinical recurrence was detected in four patients total, with two patients experiencing local recurrence and two patients experiencing distant metastasis. There were four patients who were human papillomavirus positive, as determined by surrogate p16 immunohistochemical staining. Of these human papillomavirus positive patients, one patient had local recurrence and one patient had distant metastasis. Supporting Information Table S1 summarizes primary tumor and baseline plasma mutational status.

### 3.2 Detection of potential recurrence with plasma circulating tumor DNA

Seven patients with mutations verified by SiMSen‐Seq had longitudinal blood draws following resection (Supporting Information Table S2). There were 15 post‐therapy plasma samples analyzed (range 2‐6 per patient). There was one patient with no clinical recurrence detected.

**Figure 3:**
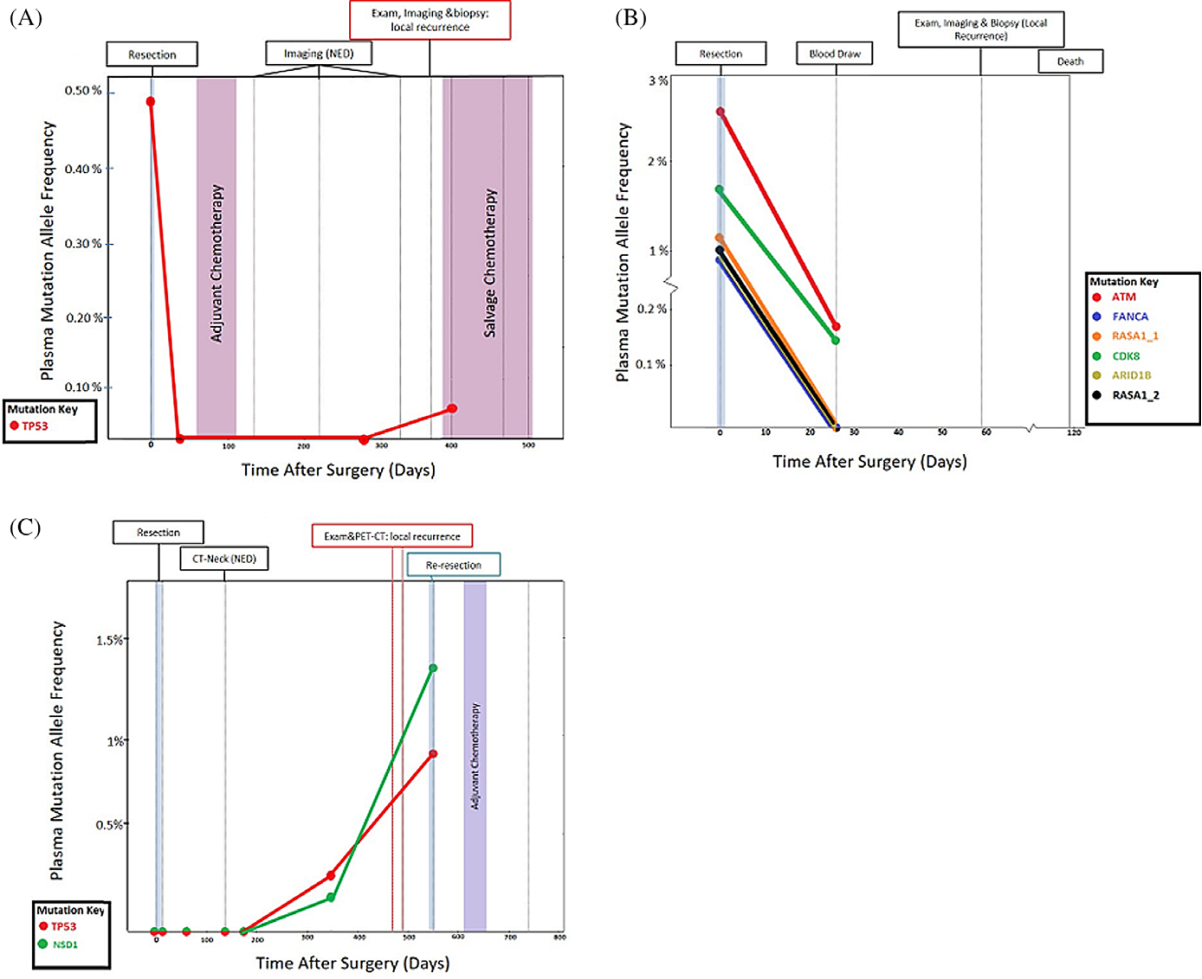
Treatment and surveillance for patients with diagnosed recurrence. (A) Patient HN01 had a TP53 tumor mutation detectable in baseline circulating tumor DNA which was not seen postoperatively. The patient was found to have no detectable recurrence on imaging and plasma until clinical and radiographic recurrence was found 384 days post‐operatively. Plasma circulating tumor DNA was positive at the next subsequent blood draw 13 days after diagnosed recurrence. (B) Patient HN02 was found to have a lack of clearance of plasma circulating tumor DNA at blood draw 3 weeks post operatively. Clinical exam, imaging, and biopsy confirmed recurrence at the next postoperative visit. (C) Patient HN04 was found to have two tumor mutations which were not detectable in baseline plasma just prior to resection, but both of which were detectable prior to recurrence diagnosed on clinical exam and imaging [Color figure can be viewed at wileyonlinelibrary.com]

There were four patients who had local recurrence of their disease. Mutations in longitudinal plasma samples were observed in three patients (Figure 3). In the remaining patient, there were mutations detected in the baseline plasma sample which were not observed 3 weeks post‐resection. A plasma sample was not available between this time and the diagnosis of clinical recurrence approximately 150 days post‐operatively. Among the three patients with mutations in longitudinal plasma samples, one patient with p16 positive stage IVA disease of the oral cavity treated with surgery and adjuvant chemoradiation therapy (HN01) had a mutation of the TP53 gene detected 1‐year post‐therapy. Plasma circulating tumor DNA was negative 10 months following resection, and detection of clinical recurrence occurred at the same time as detection with circulating tumor DNA (Figure 3A). One patient (HN04) with local recurrence presented with p16 positive, stage I (pT1N0M0) disease of the oral cavity, for which curative resection without perioperative therapy was undertaken. There were two mutations detected and verified in the tumor (NSD1, TP53), however neither of these was present in the preoperative plasma sample. The patient had no evidence of disease and no mutations observed in post‐operative plasma samples until 1‐year post‐therapy (Figure 3C). Both the NSD1 (variant allele frequency 0.21%) and TP53 (variant allele frequency 0.33%) mutations were detected in plasma 1‐year at and 1.5 years post‐operatively, with the positive plasma sample occurring 156 days prior to clinical detection of recurrence.

The second patient with local recurrence (HN02; Figure 3B) had stage IVA (pT4aN2bMx), p16 negative disease of the oral cavity that was treated with resection alone. This patient had six verified tumor mutations (ARID1B, ATM, CDK8, FANCA, and two RASA1 mutations), all of which were detected in the preoperative plasma sample. ATM (variant allele frequency 0.15%) and CDK8 (variant allele frequency 0.12%) mutations were detected in plasma taken 3 weeks post‐operatively, and the patient had clinically detectable local recurrence 22 days after plasma detection.

There were two patients whose recurrent disease manifested as distant metastasis. The first of these patients (HN05) had p16 negative, stage IVA (pT2N2bMx) cancer of the hypopharynx. The patient underwent surgical resection with adjuvant chemoradiotherapy, after which blood was drawn 6 weeks and 6 months post‐operatively. There were three mutations selected and verified in the tumor (SMARCA4, TP53, XRCC2), none of which were observed in post‐operative plasma samples. The patient developed multiple biopsy positive lung nodules clinically consistent with metastases 287 days post‐operatively. A second patient with distant recurrence (HN06) presented initially with a stage IVA (pT4aN2bMx), p16 positive tumor which was resected and treated with adjuvant chemoradiation. There were two (BCL10, TP53) tumor mutations identified in this patient, both of which were observed in the pre‐operative plasma sample. Neither of these mutations was detectable 3 weeks post‐operatively, and the patient ultimately experienced recurrence at the clavicular head 208 days post‐operatively.

## 4 DISCUSSION

The primary aim of this study was to evaluate the uses of circulating tumor DNA as a biomarker for recurrence or treatment response after surgery or chemoradiation therapy with patient‐specific tumor profiles. Our data show that circulating tumor DNA is detectable prior to treatment in all our patients with stage IV disease, but not in a single patient with p16 positive stage I disease of the oral cavity. There was a high rate of validation of selected tumor mutations (80%; 22 of 28 mutations verified). The baseline plasma circulating tumor DNA detection rate among seven patients with verified tumor mutations was 86% (six out of seven patients) with 68% (15/22) mutations detected.

The plasma circulating tumor DNA detection rate among the seven stage IV patients with verified mutations was notably higher (75%; 15/20 plasma mutations positive), and consistent with data regarding circulating tumor DNA detection in other late stage cancers.^20^ This is also consistent with previously published data in head and neck cancer showing a higher rate of plasma circulating tumor DNA detection in late stage disease.^33^ Conversely, we had only one patient with stage I disease and circulating tumor DNA was undetectable prior to treatment. Early stage cancers in previous studies were not detectable at the same rate as late stage disease, with observed plasma mutations occurring in 47%‐70% of patients.^20^, ^33^ Regarding the detectability of circulating tumor DNA amplicons themselves, our previous experience has shown increased plasma circulating tumor DNA detection when using short amplicons of fewer than 90 bp (unpublished data). Only one assay for a mutation in patient HN02 was designed with a long amplicon (95 bp) which was still detected in plasma.

Interestingly, circulating tumor DNA in previous research was more often detected in patients with metastatic disease.^20^, ^33^ In our study, both patients with distant metastases did not have detectable mutations in plasma at the time of clinical recurrence. One patient with human papillomavirus negative disease was diagnosed with multiple distant metastasis to the lung. While the clinical presentation was consistent with metastatic disease, there is no diagnostic standard for differentiating primary lung cancers from metastatic head and neck cancers in human papillomavirus negative patients. In comparison, two patients in our study with local clinical recurrence had detectable circulating tumor DNA prior to manifestation of their recurrence. One patient with early local recurrence did not have a negative circulating tumor DNA following resection despite negative resection margins, indicating that this patient may have had microscopic disease or metastatic disease despite negative margins and clearing of the appropriate nodal basins; this is a recognized and difficult clinical problem, and finding this circulating tumor DNA positivity may indicate that it holds promise in helping to determine stratification of additional therapy or closer surveillance. This would mirror findings with circulating tumor DNA in colorectal patients, where circulating tumor DNA preceded early recurrence in 80% of patients studied.^26^

Salivary and serum detection of disease have been the recent focus of innovation in the development of the liquid biopsy for head and neck cancer.^4^, ^7^, ^17^, ^18^, ^19^, ^24^, ^30^, ^33^ Most studies have evaluated plasma or salivary human papillomavirus positivity with PCR based detection methods.^7^, ^17^, ^24^ Unfortunately, human papillomavirus positivity is associated with only approximately 50% of head and neck cancer diagnoses in western countries, and only a quarter of diagnoses worldwide.^2^ It is almost exclusively in those with oral/oropharyngeal cancers, and carries a significantly improved survival and a decreased likelihood of early recurrence.^2^ To our knowledge, there has been only one other study evaluating the detection of circulating tumor DNA based on somatic mutations using a combination of sequencing and PCR techniques.^33^ While the authors of this study queried circulating and salivary human papillomavirus status in parallel to assessing for circulating and salivary somatic tumor mutations, we did not investigate plasma human papillomavirus status. Similar to our findings, the authors followed nine patients longitudinally and were able to detect circulating tumor DNA prior to clinical diagnosis of recurrence in two patients, although the nature of recurrence was not revealed.^33^ It is well demonstrated that human papillomavirus‐positive tumors and human papillomavirus‐negative tumors carry different biological characteristics and prognosis, with the latter having worse profiles for both; this underscores the importance of the current study and further differentiates it from previous research.

Our study was limited by our sample size of eight patients. While several authors have previously investigated the detection of human papillomavirus in saliva and plasma, we focused on plasma alone and identified patient‐specific mutations. We neither assessed salivary samples nor did we study the human papillomavirus status of our longitudinal plasma samples. Despite these limitations, we have added substantially to the literature of circulating tumor DNA utility in the longitudinal follow‐up of head and neck cancer patients using a unique and sensitive method of mutational detection.

### CONCLUSION

We studied the utility of circulating tumor DNA in head and neck cancer patients as a diagnostic tool for disease monitoring and recurrence. Our findings show that plasma circulating tumor DNA is detectable in late stage disease and that plasma circulating tumor DNA positivity for those who receive curative therapy may serve as a biomarker for local recurrence of disease prior to clinical manifestation. While we are limited by our modest sample size, there is a pattern of circulating tumor DNA detectability preceding local recurrence. This relationship remains under investigation, however, for patients with distant metastasis. There is a pressing need for a clinical tool to better detect recurrence early and direct the implementation of secondary treatment strategies in head and neck squamous cell carcinoma. The ability to detect early biochemical recurrence with circulating tumor DNA may lead to earlier administration of therapy and possibly more accurate prognoses in characterizing disease. Future studies with larger cohorts should characterize the pattern of circulating tumor DNA detection in local versus distant recurrence, and should aim to delineate the accuracy of salivary and plasma circulating tumor DNA positivity based on tumor location.

## Supporting information

Table S1 ‐Tumor characteristics and baseline plasma information
Table S2 ‐ Longitudinal plasma samples for eight patients with head and neck cancer following primary therapy

Table S3 ‐ Mutational assays tested and baseline plasma allele frequencies for 8 patients with Head and Neck Cancer
