# Supplementary material for: Plasma circulating tumor DNA as a potential tool for disease monitoring in head and neck cancer
Source: Head Neck. 2018 Dec 15;41(5):1351–8. doi: 10.1002/hed.25563 (PMC6467749; doi:10.1002/hed.25563)
Supplement: Supplementary file 1 — Table S1 ‐Tumor characteristics and baseline plasma information Table S2 ‐ Longitudinal plasma samples for eight patients with head and neck cancer following primary therapy [file HED-41-1351-s001.docx]

| ID | TNM at Blood Draw | AJCC Stage | p16 | Modality of Therapy | Time of Baseline Plasma | # Tumor Mutations | # Mutational Assays Designed | Assay Length | # Assays Verified in Tumor | # Assays Sequenced in Baseline Plasma | # Positive Baseline Plasma Assays |
| --- | --- | --- | --- | --- | --- | --- | --- | --- | --- | --- | --- |
| HN-01 | pT4aN2cMx | stage IVA | Positive | Surgery + Adjuvant Chemoradiation | Preoperative | 5 | 3 | Short | 3 | 3 | 1 |
| HN-02 | **pT4aN2bMx** | stage IVA | Negative | Surgery | Preoperative | 11 | 8 | Short + Long | 6 | 6 | 6 |
| HN-03 | **pT1N2bMx** | stage IVA | Negative | Surgery + Adjuvant Chemoradiation | Preoperative | 6 | 4 | Short | 4 | 4 | 1 |
| HN-04 | **pT1N0Mx** | Stage I | Positive | Surgery | Preoperative | 3 | 2 | Short | 2 | 2 | 0 |
| HN-05 | pT1N2bMx | stage IVA | Negative | Surgery + Adjuvant Chemoradiation | Preoperative | 4 | 3 | Short | 3 | 3 | 3 |
| HN-06 | pT4aN2bMx | stage IVA | Positive | Surgery + Adjuvant Chemoradiation | Preoperative | 5 | 2 | Short | 2 | 2 | 2 |
| HN-07 | cT2N3Mx | stage IVB | Positive | Chemoradiation | Preoperative | 5 | 4 | Short | 0 | 4 | 0 |
| HN-08^+^ | pT4aNxMx | stage IVA | Negative | Surgery | Preoperative | 3 | 2 | Short | 2 | 2 | 2 |

**Table S1 –Tumor characteristics and baseline plasma information**

^+^Patient presented here with a second primary tumor of the oropharynx two years following resection and adjuvant radiation for a pT2N2cM0 oral cavity tumor

| Sample | AJCC Stage | Modality of Therapy | Time of Plasma | Number Positive Plasma Assays | Mutations Selected | Clinical Recurrence | Positive Plasma prior to Clinical Recurrence |
| --- | --- | --- | --- | --- | --- | --- | --- |
| HN-01 | stage IVA | Surgery + Adjuvant Chemoradiation | Preoperative | 1 | TP53 | Local | No |
| HN-01 |  |  | 6 weeks post-therapy | 0 | None |  |  |
| HN-01 |  |  | 10 months post-therapy | 0 | None |  |  |
| HN-01 |  |  | 1-year post-therapy | 1 | TP53 |  |  |
| HN-02 | stage IVA | Surgery | Preoperative | 5 | ARID1B, ATM, CDK8, FANCA, RASA1 | Local | Yes |
| HN-02 |  |  | 3 weeks post-therapy | 2 | ATM, CDK8 |  |  |
| HN-03 | stage IVA | Surgery + Adjuvant Chemoradiation | Preoperative | 1 | TP53 | None | --- |
| HN-03 |  |  | 3 weeks post-therapy | 0 | None |  |  |
| HN-04 | Stage I | Surgery | Preoperative | 2 | NSD1, TP53 | Local | Yes |
| HN-04 |  |  | 3 weeks post-therapy | 0 | None |  |  |
| HN-04 |  |  | 2 months post-therapy | 0 | None |  |  |
| HN-04 |  |  | 4 months post-therapy | 0 | None |  |  |
| HN-04 |  |  | 6 months post-therapy | 0 | None |  |  |
| HN-04 |  |  | 1-year post-therapy | 2 | NSD1, TP53 |  |  |
| HN-04 |  |  | 1.5 years post-therapy | 2 | NSD1, TP53 |  |  |
| HN-05 | stage IVA | Surgery + Adjuvant Chemoradiation | Preoperative | 3 | SMARCA4, TP53, XRCC2 | Distant | No |
| HN-05 |  |  | 6 weeks post-therapy | 0 | None |  |  |
| HN-05 |  |  | 6 months post-therapy | 0 | None |  |  |
| HN-06 | stage IVA | Surgery + Adjuvant Chemoradiation | Preoperative | 2 | BCL10, TP53 | Distant | No |
| HN-06 |  |  | 3 weeks post-therapy | 0 | None |  |  |
| HN-08 | stage IVA | Surgery | Preoperative | 2 | RPTOR, TP53 | Local | N/A^+^ |
| HN-08^+^ |  |  | 3 weeks post-therapy | 0 | None |  |  |

**Table S2 –** **Longitudinal plasma samples for eight patients with head and neck cancer following primary therapy**

^+^Plasma sample not available at or prior to the time of diagnosis of local recurrence.
