# Supplementary material for: Plasma circulating tumor DNA as a potential tool for disease monitoring in head and neck cancer
Source: Head Neck. 2018 Dec 15;41(5):1351–8. doi: 10.1002/hed.25563 (PMC6467749; doi:10.1002/hed.25563)
Supplement: Supplementary file 2 — Table S3 ‐ Mutational assays tested and baseline plasma allele frequencies for 8 patients with Head and Neck Cancer [file HED-41-1351-s002.docx]

| Sample ID | Mutational Assays | Baseline Plasma Allele Frequency | Sample ID | Mutational Assays | Baseline Plasma Allele Frequency |
| --- | --- | --- | --- | --- | --- |
| HN-01 | TP53 | 0.57% | **HN-05** | SMARCA4  TP53  XRCC2 | 3.1%  0.77%  3.75% |
| HN-02 | ARID1B  ATM  CDK8  FANCA  RASA1  RASA1 | 0.91%  2.55%  1.6%  0.91%  1.12%  1.03% | **HN-06** | BCL10  TP53 | 4.4%  3.5% |
| HN-03 | TP53  CSMD2  SIN3A  KRAS | 0.16%  --  --  0.06% | **HN-07** | CSMD2  ANK2  FAT2  RELN | --  --  --  -- |
| HN-04 | NSD1  TP53 | Neg  Neg | **HN-08** | RPTOR  TP53 | 0.06%  0.18% |

**Table S3 – Mutational assays tested and baseline plasma allele frequencies for 8 patients with Head and Neck Cancer**
